# World diabetes day; 2013

**DOI:** 10.12861/jrip.2013.39

**Published:** 2013-10-10

**Authors:** Hajieh Shahbazian

**Affiliations:** ^1^Diabetes Research Center, Ahvaz Jundishapur University of Medical Sciences, Ahvaz, Iran

**Keywords:** World diabetes day, Type 2 diabetes, Insulin

**Implication for health policy/practice/research/medical education:**

World diabetes day was introduced by the international diabetes federation (IDF) and WHO in 1991, in response to increasing prevalence of diabetes around the world. Free screening program, public information meeting, diabetes workshop and conferences should be held by health departments, medical sciences universities and diabetes research centers in order to aware the community about this fast growing epidemic. Life style modification program should be encouraged and facilitate in order to prevent this epidemic of diabetes.

World diabetes day takes place on 14 November every year. The date was chosen because it is the birthday of Fredrick Banting who along with Charles Best is credited by the discovery of insulin in 1922.

World diabetes day was introduced by the international diabetes federation (IDF) and WHO in 1991, in response to increasing prevalence of diabetes around the world.

There are currently 366 million people with diabetes all over the world. This figure is predicted to rise to over 550 million by 2030. Diabetes is among the top 10 causes of disability resulting in life-threatening complications such as stroke, coronary heart disease, non-traumatic lower limb amputation, renal failure and blindness. Diabetes is responsible for 4.6 million deaths per year (1 death every 7 seconds) (1).

One in 10 adults has diabetes. Although the global average prevalence of diabetes is about 10%, up to one third of people in some Pacific Island countries suffered from diabetes (2).

Prevalence of diabetes in different part of Iran reported to be between 3 to 16.3% (3). Overall, there are more than 2 million patients with diabetes in Iran (4).

Diabetes is a fast growing chronic disease and unless action is taken now, it expected to overtake heart disease and cancer to become the largest cause of disability and premature death in future. The good news is that type 2 diabetes can be prevented through positive life style changes. In fact, the risk of developing type 2 diabetes can be reduced up to 60% by maintaining a healthy weight, following a healthy diet and being physically active (5).

Early detection is one of important steps in managing diabetes, because unfortunately about 50% of patient with diabetes are unaware of their disease (6). Diagnosis and appropriate management is paramount. Many studies showed that good management of diabetes can greatly reduce its complications or delay or prevent them (7,8).

World diabetes day is an ideal opportunity to focus attention on diabetes. Some activities should be organized to aware the community about the risk factors, screening, diagnosis and treatment of diabetes. Radio and television program, newspaper and magazine articles may be very helpful. Free screening program, public information meeting, diabetes workshop and conferences should be held by health departments, medical sciences universities and diabetes research centers in order to aware the community about this fast growing epidemic. Life style modification program should be encouraged and facilitate in order to prevent this epidemic.

## Author’s contribution

HS is the single author of the manuscript.

## Conflict of interests

The author declared no competing interests.

## Ethical considerations

Ethical issues (including plagiarism, informed consent, misconduct, double publication and redundancy) have been completely observed by the authors.

## Funding/Support

None declared.
